# Chemokine Therapy in Cats With Experimental Renal Fibrosis and in a Kidney Disease Pilot Study

**DOI:** 10.3389/fvets.2021.646087

**Published:** 2021-03-04

**Authors:** Julie Bennington, Shannon Lankford, Renata S. Magalhaes, Douglas Shankle, Jason Fanning, Cucu Kartini, Irma Suparto, Winda Kusumawardhani, M. ArRaniri Putra, Silmi Mariya, Gopal Badlani, J. Koudy Williams

**Affiliations:** ^1^Wake Forest Institute for Regenerative Medicine, Winston-Salem, NC, United States; ^2^Department of Health and Exercise Science, Wake Forest University, Winston-Salem, NC, United States; ^3^Praktek Dokter Hewan Bersama Joint Veterinary Practice, Sunter, Indonesia; ^4^Primate Research Center, Institut Pertanian Bogor, Bogor Agricultural University, Bogor, Indonesia; ^5^Department of Urology, Wake Forest Baptist Health, Winston-Salem, NC, United States

**Keywords:** chemokine CXCL12, stromal cell-derived factor (SDF)-1α, local intra-renal injection, tubulo- interstitial fibrosis, ischemia/reperfusion injury, histomorphometry of collagen fibers

## Abstract

**Background:** Chronic tubulointerstitial fibrosis is a common final pathway leading to end stage kidney disease in cats and has no effective treatment. The use of cell-based molecules to treat kidney fibrosis may be a promising approach. The objectives were to test the effects of intra-renal chemokine CXCL12 injection in a pre-clinical cat model of unilateral ischemia/reperfusion (I/R)-induced kidney fibrosis and then, within a clinical pilot study, test the safety/feasibility of CXCL12 injection in cats that might have early chronic kidney disease (CKD).

**Methods:** *Pre-clinical*: Thirty cats received intra-renal injection of 100, 200, or 400 ng of recombinant human CXCL12, or sterile saline, into the I/R kidney 70 days post-injury, or were non-injured, non-injected controls (*n* = 6/group). Kidney collagen content was quantified 4 months post-treatment using Masson's Trichrome and Picrosirius Red (PSR) stained tissues. In a separate study (*n* = 2) exploring short-term effects of CXCL12, 200 ng CXCL12 was injected into I/R kidneys and then harvested either 30 min (*n* = 1) or 1 month (*n* = 1) post-injection. Kidney concentrations of CXCL12, matrix metalloproteinase 1 (MMP-1), and lysyl oxidase-like enzyme 2 (LOXL-2) were quantified *via* ELISA. *Clinical Pilot*: 14 client-owned cats with potential early kidney disease received a single-treatment, bilateral intra-renal injection of 200 ng CXCL12 (*n* = 7), or received no injection (*n* = 7). Blood/urine samples were collected monthly for 9 months to assess renal function and CKD staging.

**Results:** *Pre-clinical*: I/R increased the affected kidney collagen content, which both mid and high doses of CXCL12 restored to normal (*ps* < 0.05 vs. untreated). I/R increased collagen fiber width, which both mid and high doses of CXCL12 restored to normal (*p* < 0.001 vs. untreated). Early changes in kidney MMP-1, associated with collagen breakdown, and subsequent decreases in LOXL-2, associated with collagen cross-linking, in response to CXCL12 treatment may contribute to these findings. *Clinical Pilot*: Bilateral intra-renal injection of CXCL12 using ultrasound guidance in cats with CKD was feasible and safe in a general practice clinical setting with no obvious side effects noted during the 9-month follow-up period.

**Conclusions:** Intra-renal injection of CXCL12 may prove to be an effective treatment for kidney fibrosis in cats with CKD. Additional mechanistic and clinical evaluations are needed.

## Introduction

Chronic kidney disease (CKD) affects ~30% of cats age 15 years or older with the prevalence increasing with age (1–5). Current treatments include pharmaceutical therapies and dietary management to slow disease progression, increase longevity, and improve quality of life, but alternatives are needed.

The underlying pathology leading to End Stage Renal Disease (ESRD) is chronic tubulointerstitial fibrosis (6–16). Kidney tissue regenerative therapies designed to restore functional kidney units have been tested in several studies. These include various combinations of bone marrow and adipose-derived mesenchymal stem cells and stromal vascular fraction therapies and different routes of administration (intravenous, intra-renal, and intra-arterial) (17–23). While modestly effective, the clinical applicability of these cell therapies are limited because they are expensive, time consuming, and require advanced cell processing capabilities not available in a general practice setting.

As an alternative, we tested a chemokine (CXCL12), produced by cells to promote tissue regeneration in damaged tissues (24–32). The recombinant human form is commercially available and inexpensive. Injection of recombinant human CXCL12 protein has been shown to reduce fibrosis in rodent models of chronic kidney disease (28), uterine fibrosis (30), stress urinary incontinence (33, 34), and non-human primate models of intrinsic sphincter deficiency (35, 36) and erectile and urinary dysfunction post-radical prostatectomy (37), but its effects on collagen content and collagen fiber histomorphometry have not been tested in cat models of chronic renal fibrosis.

**Figure 1:**
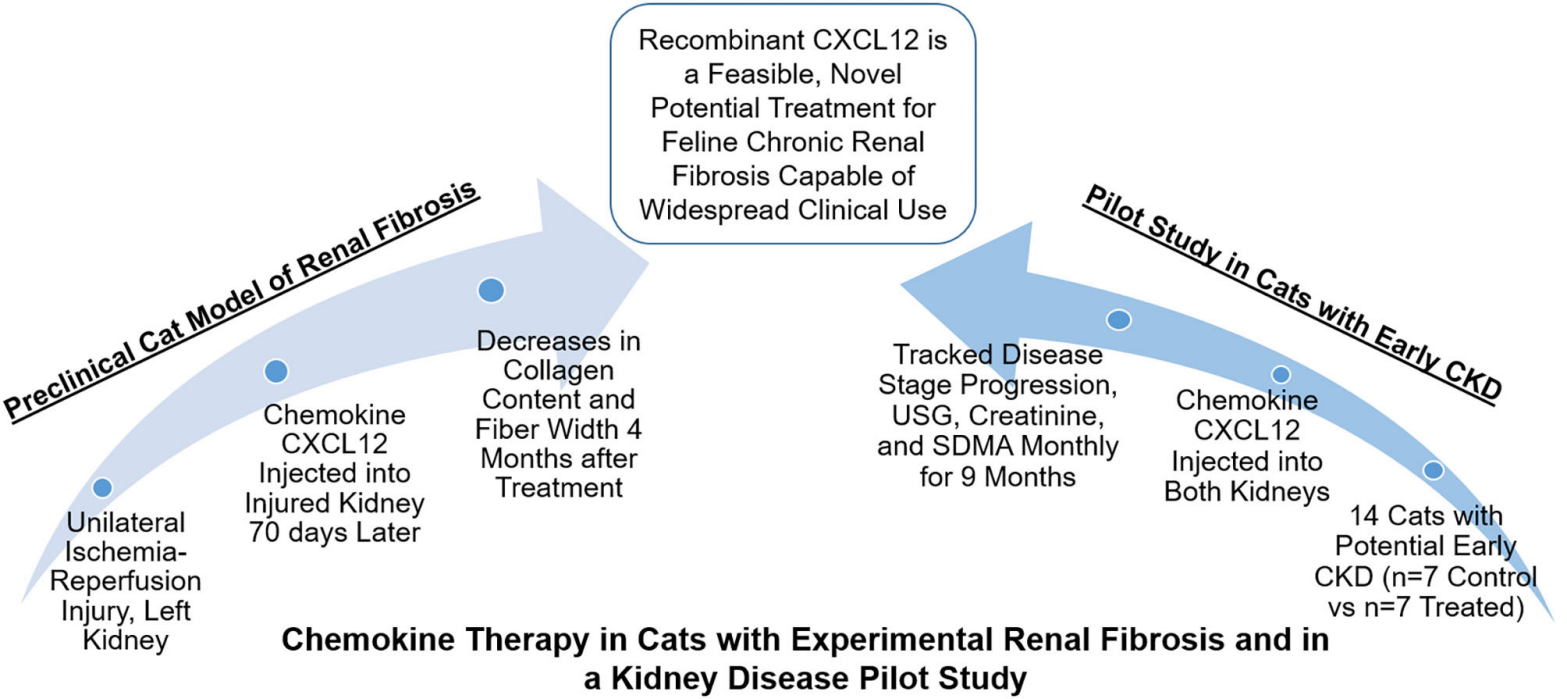
Visual Abstract Summary Diagram.

Thus, the goal of this study was to test the effects of the chemokine CXCL12 in chronic renal fibrosis in cats. We first tested the dose-effects of CXCL12 in cats with unilateral ischemia/reperfusion (I/R)-induced interstitial fibrosis (38). Then in a pilot study, we tested the feasibility of ultrasound-guided intra-renal CXCL12 injection in a general practice setting in cats with potential early CKD (Figure 1: Visual Abstract Summary Diagram).

## Materials and Methods

### Animals—Pre-clinical Study

**Figure 2:**
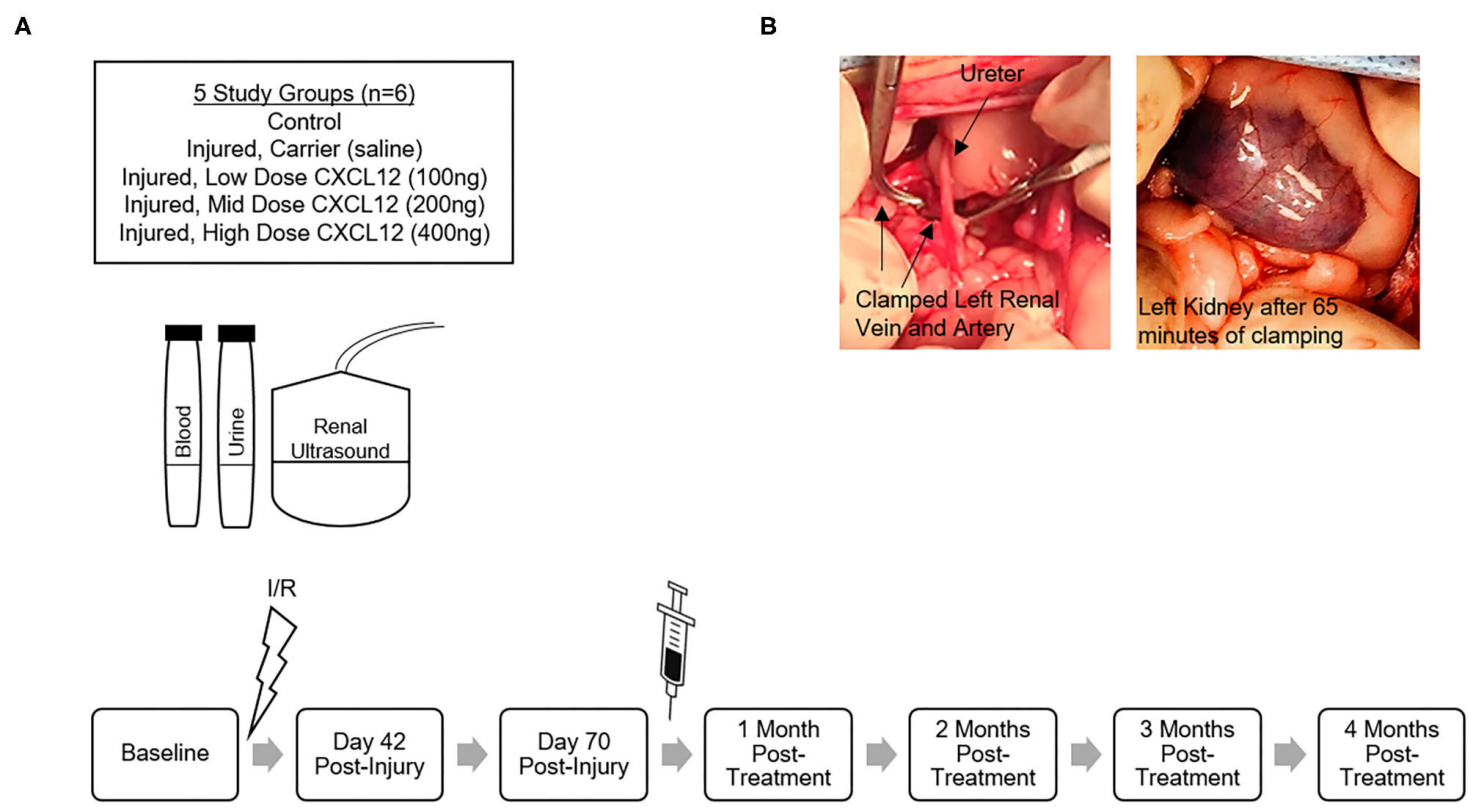
**(A)** Schematic of pre-clinical study design and timeline using 5 groups of cats to test the effects of intra-renal injection of 100, 200, or 400 ng (*n* = 6/dose) of CXCL12, or sterile saline carrier (*n* = 6), administered 70 days after I/R-induced renal fibrosis. Six cats in a control group were uninjured and not injected. Baseline blood/urine renal function tests and ultrasounds were performed at the time of I/R Injury, Day 42, Day 70 (Treatment injection), and then monthly for 4 months post-injection. **(B)** Intraoperative images during I/R injury surgery with vascular clamps occluding left renal artery and left renal vein and purple discoloration of the kidney. Clamps remained in place for 65 min and then were released restoring renal blood flow that was confirmed using a Doppler probe to detect renal artery pulsation.

Cats with surgically-produced ischemia/reperfusion (I/R)-induced renal fibrosis were used to test the effects of intra-renal injection of 100, 200, or 400 ng (*n* = 6/dose) of CXCL12, or saline carrier (*n*=6), on renal structure in the affected kidney (Figure 2A). Six cats in a control group were anesthetized but uninjured and not injected. They were all fed maintenance diets (Laboratory Feline Diet, LabDiet, St. Louis, MO). Baseline blood/urine renal function tests and ultrasounds were performed at the time of I/R Injury, day 42, day 70 (Treatment injection), and then monthly for 4 months post-injection. At 4 months post-injection, necropsies were performed and tissues harvested for histological evaluation.

Two cats were used in a small, separate study exploring early changes in local kidney concentrations of CXCL12, collagenase, and a collagen cross-linking enzyme. Using the Mid dose (200 ng) of CXCL12, one cat with a 5-month I/R injury was necropsied 30 min post-injection. The other cat with a 4-month I/R injury was necropsied 1 month post-injection. Tissues were harvested and flash frozen in cryotubes with liquid nitrogen then stored at −80°C for ELISA assay.

Study design and animal procedures were approved by the Institutional Animal Care and Use Committee (IACUC) at the Wake Forest University School of Medicine. The pre-clinical study used 32 healthy, purpose-bred, 8-month-old female cats acquired from Liberty Research, Inc. (Waverly, NY). Cats were examined by the Wake Forest University Animal Resources Program (ARP) veterinary staff within 3 days of arrival, screened for intestinal parasites, microchipped, and vaccinated with a Rabies vaccine. Cats were vaccinated by the vendor with core vaccines (Feline Rhinotracheitis Virus/Herpesvirus 1, Feline Calicivirus, Feline Panleukopenia) prior to arrival. Cats were quarantined for 3 weeks and deemed healthy by the ARP staff veterinarian prior to release for this study.

### Cat Model of Renal Fibrosis—Pre-clinical Study

A slight modification to the protocol for producing interstitial renal fibrosis described by Schmiedt et al. (38) was used for these studies (the duration of ischemia was increased to 65 min, instead of 60 min, before reperfusion). Cats were fasted and pre-medicated with buprenorphine, ketamine, midazolam, and glycopyrrolate. Intravenous (IV) catheter and endotracheal tube were placed, and Lactated Ringers Solution (LRS) was administered IV. Anesthesia was maintained using isoflurane in oxygen. Famotidine and maropitant citrate were also administered parenterally. Cats were shaved for renal ultrasound and surgically prepared for sterile ventral laparotomy. Both kidneys were exposed and visually inspected, and vasculature of the left kidney was isolated. Vascular clamps were placed across the renal artery first and renal vein second, and purple discoloration of the kidney was observed as visual evidence of hypoxia (Figure 2B). Clamps remained in place for 65 min and then were released restoring renal blood flow that was confirmed using a Doppler probe to detect pulsation of the renal artery. The incision was closed in three layers, cats were recovered in their cages, and appropriate analgesics (e.g., robenacoxib, buprenorphine) were administered as described in the approved IACUC Protocol.

### Treatment Injection—Pre-clinical Study

At day 70 after the I/R procedure, cats were fasted and pre-medicated with buprenorphine, ketamine, and midazolam. Anesthesia was maintained using isoflurane in oxygen via mask. LRS was administered subcutaneously. Bilateral flanks were shaved, and renal ultrasound was performed in lateral recumbency for each kidney to assess baseline *in vivo* kidney size (Acclarix AX8 Portable Ultrasound System, Edan USA, San Diego, CA). The left flank was prepared for aseptic ultrasound-guided intra-renal injection. Using a sterile-covered linear probe (L10-4Q) to obtain a longitudinal view of the left kidney, a single injection was administered into the middle of the left kidney mid-cortex level with a sterile 27-gauge needle to deliver .25ml total volume of sterile saline or saline plus CXCL12 (Recombinant Human SDF-1α (CXCL12), Catalog #300-28A, PeproTech Inc., Rocky Hill, NJ) that was reconstituted and aliquoted sterilely using a 0.45 um filter. All injections were performed by one investigator using ultrasound guidance.

In the separate, small study (*n* = 2) exploring early changes in local kidney concentrations of CXCL12, collagenase, and a collagen cross-linking enzyme, similar procedures were used to administer a single, intra-renal 200 ng CXCL12 injection at 5 months post-I/R injury into the affected kidney in one cat, and at 4 months post-I/R injury into the affected kidney in the other cat.

### Necropsy and Evaluation of Tissues—Pre-clinical Study

Four months after treatment, cats were sedated, heparinized, and euthanized using sodium pentobarbital in accordance with American Veterinary Medical Association Guidelines. One liter of saline was infused into the left cardiac ventricle exiting the lanced caudal vena cava to remove residual blood. Gross pathology was documented and photographed. Both kidneys were removed, weighed, photographed, and measured using calipers with renal capsules intact. Both kidneys were incised transversely into four quarter sections. Two sections were immersed in 4% paraformaldehyde for 72 h then transferred to 70% ethanol prior to paraffin embedding. The remaining two sections were incised transversely into three sections and flash frozen in 3 cryotubes with liquid nitrogen then stored at −80°C. Remaining harvested tissues were immersed in 10% neutral buffered formalin (NBF) for 48 h then transferred to 70% ethanol prior to paraffin embedding. Paraffinized left kidney tissue was sectioned into 5 um slices using a microtome, and histology slides were stained with Masson's Trichrome stain (#HT15, Sigma-Aldrich Inc., St. Louis, MO) and Picrosirius Red (PSR) (#24901, PolySciences, Warrington, PA) for collagen content analyses.

In the small study exploring changes in local kidney concentrations of CXCL12, collagenase, and a collagen cross-linking enzyme, similar procedures were followed except that cats were not heparinized, infusion with one liter of saline was not performed, and necropsy was performed 30 min post-treatment in one cat and 1 month post-treatment in the other cat.

### Imaging, Quantitative Analysis of Fibrosis, Collagen Fiber Analysis, and ELISAs—Pre-clinical Study

**Figure 3:**
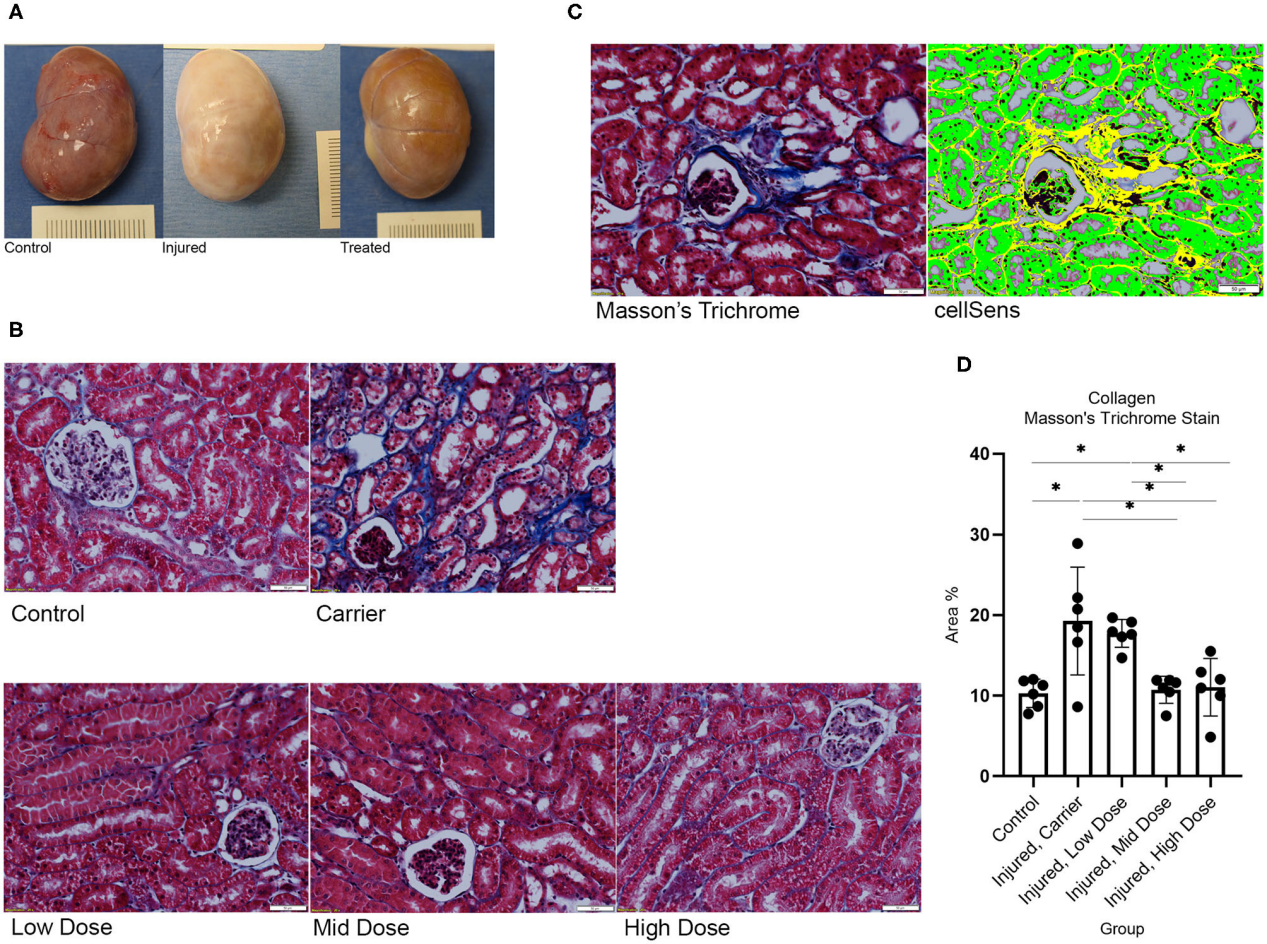
**(A)** Gross necropsy of left kidney for Control, I/R injured, and CXCL12 treated. I/R resulted in renal fibrosis (white discoloration) that was restored with treatment. **(B)** Histology of left kidney CMJ stained with Masson's Trichrome under 200× magnification for each study group. I/R (Carrier) increased collagen accumulation, stained blue. CXCL12 Treatment restored I/R-induced changes in collagen in a dose-dependent manner. **(C)** Masson's Trichrome stain image and cellSens Imaging Software-generated image used to calculate Area % of collagen (yellow). **(D)** One-way ANOVA for Area % of positive staining for collagen for each group on Masson's Trichrome stain showed a significant group effect, *F*_(4,25)_ = 8.408, *p* < 0.01. *n* = 6/group. Values are individual dot plot and mean ± SD. *p < 0.05.

**Figure 4:**
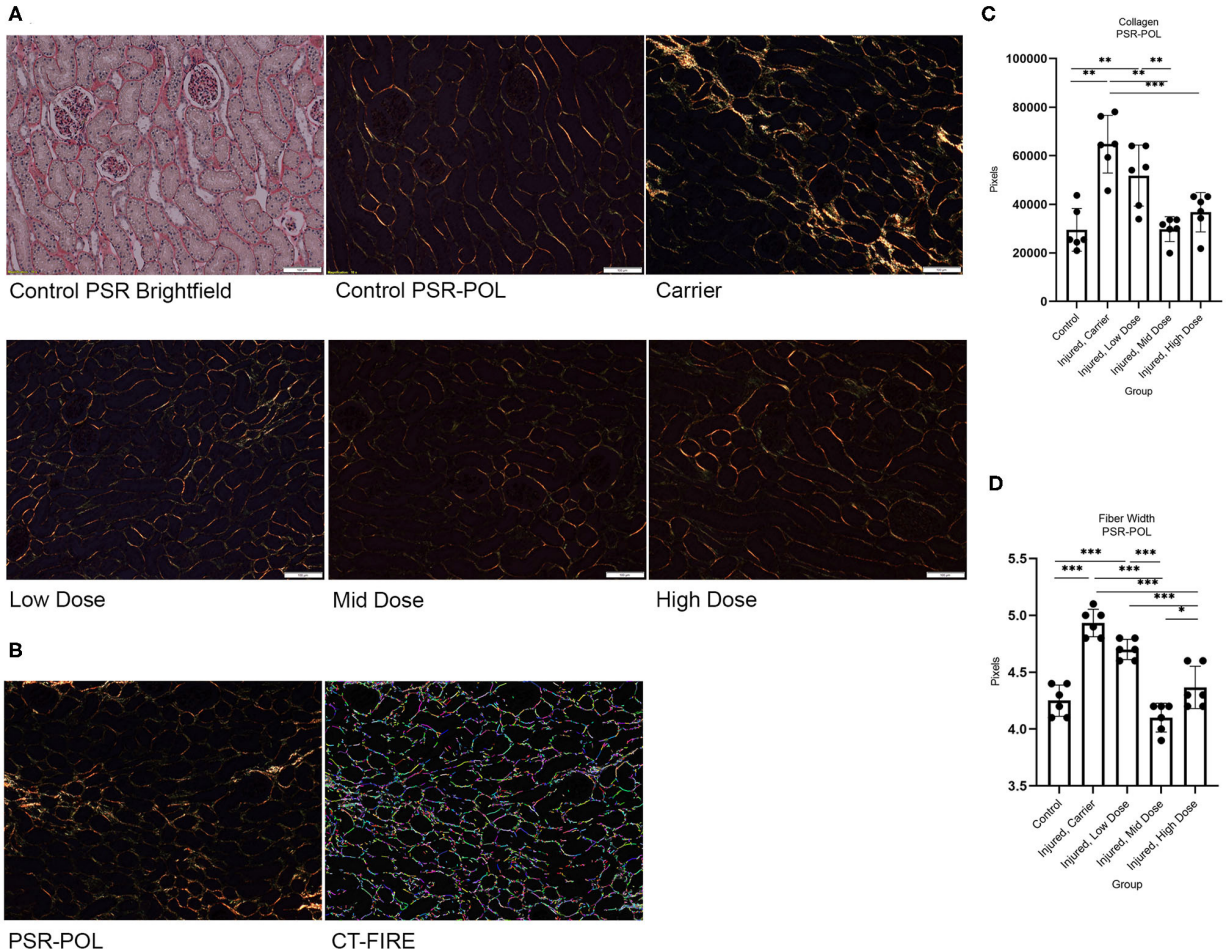
**(A)** Histology of left kidney CMJ stained with PSR and PSR-POL under 100× magnification for each study group. I/R (Carrier) increased birefringent collagen fiber signal. CXCL12 Treatment restored I/R-induced changes in birefringent collagen fiber signal in a dose-dependent manner. **(B)** PSR-POL image used to calculate total collagen in pixels and segmentation software CT-FIRE-generated image used to quantify collagen fiber length and width. **(C)** One-way ANOVA for collagen fiber width for each group on PSR-POL showed a significant group effect, *F*_(4,25)_ = 36.634, *p* < 0.001; and **(D)** One-way ANOVA for total birefringent collagen signal in pixels for each group on PSR-POL showed a significant group effect, *F*_(4,25)_ = 15.165, *p* < 0.001. *n* = 6/group. Values are individual dot plot and mean ± SD. *p < 0.05; **p < 0.01; ***p < 0.001.

Images of histology sections were taken using the Olympus BX63 microscope (Olympus, Center Valley, PA) with an Olympus DP80 camera (Olympus). Ten randomly selected, evenly spaced images were taken at 200× magnification (for Masson's Trichrome stain) and 100× magnification under the polarized light filter (for PSR stain) of each left kidney renal cortico-medullary junction (CMJ) that included at least one glomerulus but excluded large blood vessels and processing artifacts (e.g., wrinkles, tears). For tissues stained with Masson's Trichrome stain, quantitative analysis of fibrosis was performed using the Olympus cellSens dimension software (v.1.16), Count and Measure application, to calculate Area % of Collagen (Figure 3C). The results of 10 images were averaged for each cat (*N* = 30) for further analysis. For tissues stained with Picrosirius Red under polarized light (PSR-POL), two collagen fiber analyses were performed: (1) Collagen fiber quantification for length and width using the segmentation software CT-FIRE (Laboratory for Optical and Computation Instrumentation, University of Wisconsin) (39–45) (Figure 4B); and (2) Hue analysis for level of collagen bundling and total collagen quantification using a MATLAB script (R2020a, MathWorks, Natick, MA) (43, 44, 46). Results of 10 images were averaged for each cat (*N* = 30) for further analysis.

In the small study exploring early changes in local kidney concentrations of CXCL12, collagenase, and a collagen cross-linking enzyme, ELISAs were performed in duplicate using the supernatant collected from 50 mg frozen kidney tissue homogenized in Pierce RIPA Lysis and Extraction Buffer (Catalog #89900, ThermoFisher Scientific, Waltham, MA) to detect and quantify concentrations of human CXCL12 (Human SDF-1α Standard ABTS ELISA Development Kit, Catalog #900-K92, Lot #0717092, PeproTech Inc., Cranbury, NJ), matrix metalloproteinase 1 (MMP-1) (Nori Feline MMP-1 ELISA Kit, Catalog #GR188162, Genorise Scientific, Inc., Glen Mills, PA), and lysyl oxidase-like enzyme 2 (LOXL-2) (Human LOXL-2 ELISA Kit, Catalog #ab213808, Abcam, Cambridge, MA).

### Clinical Pathology—Pre-clinical Study

Blood and urine samples were submitted to IDEXX Laboratories for complete blood count, serum biochemical analysis, including renal biomarker symmetric dimethylarginine (SDMA) (47–50), and urinalysis.

### Statistical Analyses—Pre-clinical Study

The effect of treatment on total collagen and collagen fiber width was evaluated for each group using a one-way ANOVA for approximately normally distributed data. Differences between groups were considered statistically significant at *p* < 0.05. Bonferroni-corrected *post-hoc*-tests were used to evaluate multiple comparisons. Results are shown as Mean ± SD. The following assumptions were checked for a one-way ANOVA: no significant outliers on visualization of boxplots, normality using the Shapiro-Wilk test for normality, and homogeneity of variances using Levene's-test of equality of variances. Winsorization was used to retain any extreme outliers. For violations of normality on Shapiro-Wilk, z-scores for skewness and kurtosis were checked to be within ± 2.58 standard deviations. All statistical analyses were performed using IBM SPSS Statistics (IBM Corp., Armonk, NY).

Given the small sample size of the study exploring early changes in local kidney concentrations of CXCL12, collagenase, and a collagen cross-linking enzyme, only descriptive summary statistics were performed.

### Animals—Pilot Study

The pilot study was done in collaboration with veterinarians at the PDHB Joint Veterinary Practice in Indonesia. Fourteen geriatric, client-owned cats with potential early kidney disease, based on physical exam, history of weight loss, polyuria/polydipsia with urine specific (USG) gravity <1.035, were divided into Control (no injection) vs. Treatment (*n* = 7/group). The inclusion criteria included: (1) Age 7 years or older; (2) Negative Feline Leukemia Virus (FeLV) and Feline Immunodeficiency Virus (FIV) test; and (3) Overall good health and mobility. Cats who met these inclusion criteria were placed in the Treatment group early in the study and, as such, were not randomized. Exclusion criteria were the presence or history of: (1) Polycystic kidney disease; (2) Urolithiasis (renal and ureteral); and (3) Respiratory disease requiring treatment (in Indonesia, feline upper respiratory infections are oftentimes highly virulent, pathogenic, and debilitating). All cats were treated with selamectin and praziquantel, and significant comorbidities (e.g., flea allergy dermatitis and stomatitis) were treated and resolved prior to treatment injections. Two cats in the Control group (Cats 5 and 6) were previously diagnosed with cardiomegaly (suspected hypertrophic cardiomyopathy) and considered stable with diltiazem and amlodipine. Baseline blood/urine renal function tests and ultrasounds were performed prior to injections and repeated monthly (blood/urine) and quarterly (renal ultrasound) for 12 months. All cats were provided with Royal Canin Renal Special prescription diet, and two Treatment group cats (Cats 1 and 2), residents of the clinic, were fed the prescription diet exclusively with no other therapies. All other cats were permitted to receive subcutaneous fluid therapy and amlodipine, if needed. Anti-proteinuric treatment, such as benazepril, is not readily available in Indonesia; human formulations of telmisartan and enalapril are available but were not needed. Although some cats were indoor only, it is common for cats in Indonesia to roam freely indoor and outdoor. Owners of the participating cats were thoroughly informed of the scope, objectives, expectations, and potential risks and benefits of the procedures to be performed in this pilot study. All clients were willing participants, signed consent forms, and were not blinded to treatment group.

### Treatment Injections—Pilot Study

Before injections, Treatment group cats were fasted and pre-medicated with diazepam IV. IV catheters were placed, and LRS was administered IV. Propofol was used for anesthesia induction, and anesthetic plane was maintained using isoflurane in oxygen via mask or endotracheal tube. Bilateral flanks were shaved, and renal ultrasound was performed in lateral recumbency for each kidney. Each flank was prepared for aseptic ultrasound-guided intra-renal injection. Using a sterile-covered convex probe to obtain a longitudinal view of the kidney, a single injection was administered into the middle of each kidney mid-cortex level using a sterile 27-gauge needle to deliver 0.25 ml of sterile saline plus 200 ng CXCL12 (Recombinant Human SDF-1α (CXCL12), Catalog #300-28A, PeproTech Inc., Rocky Hill, NJ) that was reconstituted and aliquoted sterilely using a 0.45 um filter. All treatment injections were performed by the same two investigators who were trained in the technique used in the I/R study.

### Clinical Pathology—Pilot Study

Blood and urine samples for complete blood count, serum biochemical analysis, and urinalysis were submitted to the Animal Laboratory Sunter, Indonesia for evaluation. Blood samples were also submitted to the Microbiology and Immunology Laboratory, Primate Research Center, Institut Pertanian Bogor, Bogor Agricultural University, Indonesia for SDMA (symmetric dimethylarginine) evaluation using a canine/feline SDMA ELISA kit in duplicate (#TRM-594, Biovet, Inc., St-Hyacinthe, QC, Canada). SDMA was measured over the length of the experiment but not used to determine CKD Stage. For the purpose of this study, CKD Stage was defined as follows: 1) Stage 1: urine specific gravity (USG) <1.035 and serum Creatinine <1.6 mg/dL; 2) Stage 2: USG <1.035 and serum Creatinine 1.6–2.8 mg/dL; and 3) Stage 3: USG <1.035 and serum Creatinine 2.9–5 mg/dL. A theoretical Stage 0 was formulated to denote USG 1.035 or higher and serum Creatinine <1.6 mg/dL.

### Statistical Analyses—Pilot Study

Given the small sample size and missing data points, only descriptive and summary statistics were calculated, to include median and range.

## Results

### Pre-clinical Study

I/R resulted in renal fibrosis, tubulointerstitial collagen accumulation, and widening of collagen fibers in the affected kidney. I/R significantly increased collagen content (*p* < 0.05 vs. Control) and collagen fiber width (*p* < 0.001 vs. Control) in the affected kidney. Injection of recombinant CXCL12 treatment attenuated these injury-induced changes in collagen (Figure 3A: Gross necropsy) in a dose-dependent manner (Figure 3B: Masson's Trichrome stain and Figure 4A: PSR-POL). Both the Mid and High doses of CXCL12 restored kidney collagen in I/R kidneys (*p* < 0.05 vs. untreated) as measured with Masson's Trichrome stained tissue sections (Figure 3D). This treatment effect on kidney collagen was also validated on Picrosirius Red under polarized light (*p* < 0.01 vs. untreated) (Figure 4C). Furthermore, CXCL12 treatment also restored (reduced) collagen fiber width (*p* < 0.001 vs. untreated) (Figure 4D).

**Figure 5:**
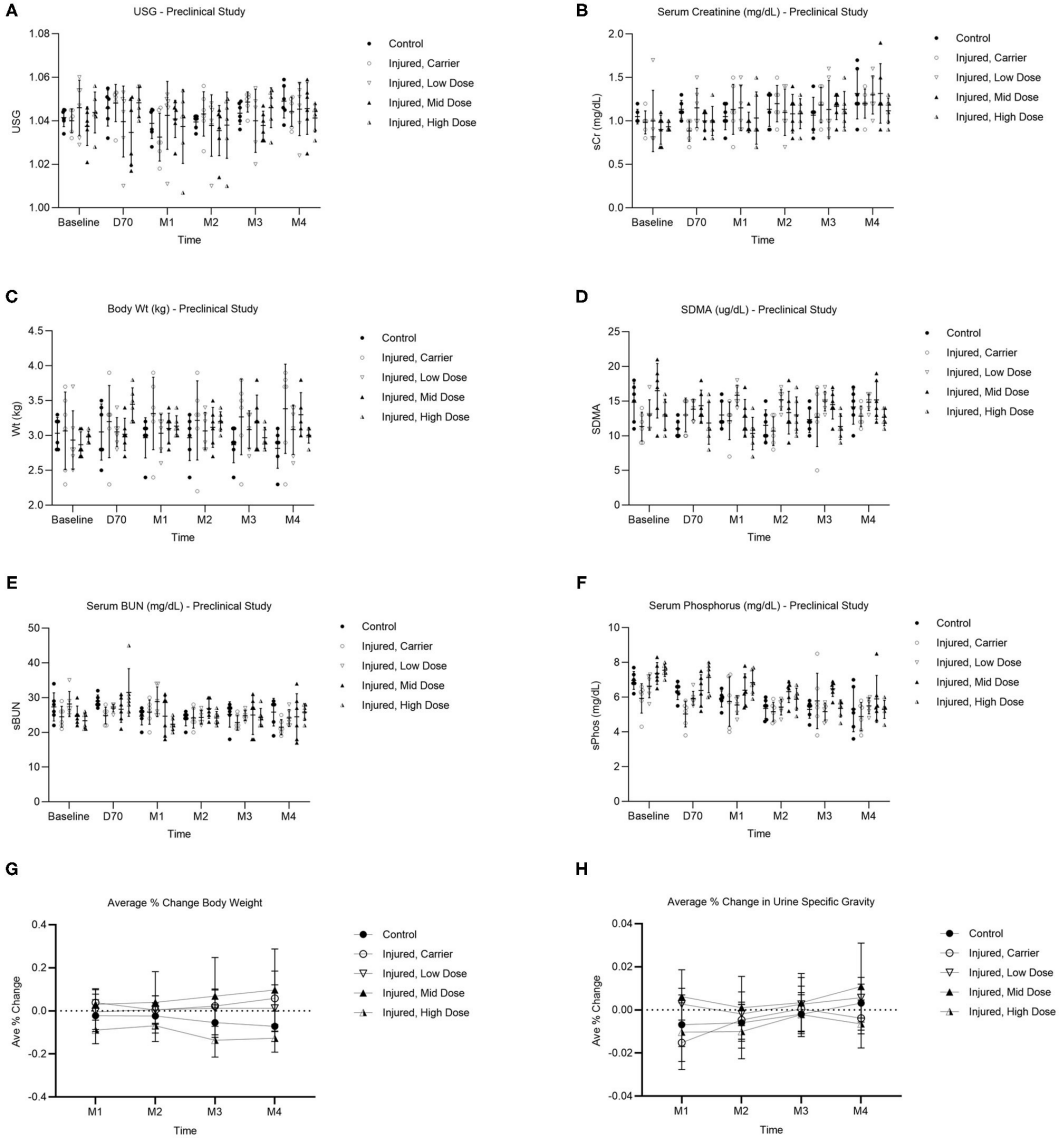
Pre-clinical study clinical pathology results of renal function over time (Baseline, Day 70, Month 1, Month 2, Month 3, Month 4) for each group depicted as dot plot and mean ± SD showing: USG **(A)**, serum creatinine **(B)**, body weight **(C)**, SDMA **(D)**, serum BUN **(E)**, and serum phosphorus **(F)**. Average % change in body weight (kg) **(G)** and USG **(H)** over time (Month 1, Month 2, Month 3, Month 4) for each group when compared to day 70 post-injury (i.e., maximum injury), just prior to treatment injection. *n* = 6/group. Values are mean ± SD.

Analysis of renal function clinical pathology endpoints over time detected no significant differences among groups. Due to the unilateral injury of our model (and subsequent compensation by the uninjured kidney), no significant differences in renal function were detected among the groups (Figures 5A–F). Changes in body weight and urine specific gravity (USG) for all groups were measured by calculating Average % change from Baseline evaluated at 1, 2, 3, and 4 months post-Treatment injection (Figures 5G,H), using Day 70 post-injury as Baseline (maximum injury), with no significant differences among groups. Supplementary Tables with raw data are also provided in the Pre-clinical Supplementary Material section.

Ultrasound-guided intra-renal injection of chemokine therapy was safe and feasible in this model. The procedure duration for bilateral renal ultrasound and unilateral intra-renal injection was <1 h. No adverse reactions (e.g., anesthetic complication, infection, immune reaction) were observed during or after the procedure. Route of administration was minimally invasive and required general practitioner-level training and equipment.

Early changes in kidney concentrations of MMP-1, associated with collagen breakdown, and LOXL-2 concentrations, associated with collagen cross-linking, were measured in the I/R-injured kidneys in response to CXCL12 Treatment (Supplementary Figure 1 in Pre-clinical Supplementary Material section).

### Pilot Study

Descriptive statistics of renal function clinical pathology endpoints are provided in Supplementary Table 1 in the Pilot Supplementary Material section. Prior to treatment, the Control group included 6 Stage 2 and 1 Stage 1 cats, and the Treatment group 5 Stage 2 and 2 Stage 1 cats. At the pilot study end, 3 Stage 2 cats and 1 Stage 1 cat in the Control group increased in disease stage (Worse), and 3 Stage 2 cats decreased (Improved) (Table 1). In the Treatment group, 3 Stage 2 cats decreased in disease stage (Improved) at the pilot study end, and 2 Stage 2 cats and 2 Stage 1 cats maintained the same stage (No Change) (Table 1). None of the treated cats advanced to a higher stage of disease at the end of the pilot study. The sum of the changes in CKD stage compared to baseline was calculated monthly for each group (Supplementary Figure 1A, Pilot Supplementary Material).

**Table 1:** Summary of CKD stage progression and corresponding number of cats at pilot study end.

	**Worse**	**No change**	**Improved**
Control	4	0	3
Treatment	0	4	3

Changes in urine specific gravity, serum creatinine, and SDMA with CXCL12 treatment were measured. Number of cats able to concentrate urine to 1.035 or higher is shown in Supplementary Figure 1B, Pilot Supplementary Material, with n specified for each group, Control (C) and Treatment (T), at each time point. Number of cats with creatinine <1.6 mg/dl (Supplementary Figure 1C, Pilot Supplementary Material) and % change in serum creatinine when compared to baseline (Supplemental Figure 1D, Pilot Supplementary Material) were calculated for both groups. Number of cats with SDMA 14 ug/dl or less (Supplementary Figure 1E, Pilot Supplementary Material) and % change in SDMA when compared to baseline (Supplementary Figure 1F, Pilot Supplementary Material) were calculated for both groups. Supplementary Tables with raw data are also provided in the Pilot Supplementary Material section.

We were unable to perform statistical analyses with significance due to missing data points resulting from the inherent challenges of collecting urine samples from client-owned cats and ending the pilot study 3 months early due to inconsistent adherence to study parameters. One owner from the Control group cats moved to a different country after 6 months. One cat from each group also developed severe respiratory symptoms requiring treatment near the end of the study, and subsequent data points were excluded.

## Discussion

The major findings of this study were: (1) Injection of recombinant CXCL12 reduced the formation of collagen and reduced collagen fiber width in a pre-clinical cat model of I/R-induced tubulointerstitial fibrosis; and (2) Kidney injection of recombinant CXCL12 was safe and feasible as local treatment in cats with early stages of CKD.

Tubulointerstitial fibrosis is the underling tissue pathology that best correlates with declining renal function (10, 11, 16, 51). Therefore, the main goal of this study was to utilize a treatment that would reduce the amount of interstitial fibrosis and then test feasibility of this treatment in a pilot study. We chose intra-renal injection of recombinant CXCL12 as our treatment. CXC motif chemokine 12 (CXCL12) is expressed in many tissues and cells. It is released mainly by mesenchymal stem cells and damaged tissues in the regenerative microenvironment (52, 53). Through a receptor (CXCR4) mechanism, CXCL12 has a major role in cell trafficking and homing of progenitor cells to sites of injury and in enhancing cell survival once at the injury site. There are numerous reports of this regenerative capacity in various tissues (28, 30, 33–37). We have previously used CXCL12 as a local treatment to stimulate regeneration of urogenital tissues (34, 36, 37).

There are published studies that seem to contradict our results and dispute the beneficial effects of CXCL12 on kidney regeneration (54), renal function and fibrosis (55, 56), and collagen synthesis in different tissues and organs. These studies report inflammatory effects of serum CXCL12 in various disease states (57, 58), to include malignancy (59), and that the CXCL12/CXCR4 mechanism may be directly involved in renal carcinoma (59). Additionally, other *in vitro* cell culture studies report CXCL12/CXCR4 axis-mediated kidney pathology and activation of collagen synthesis (56, 60, 61). However, these studies focus on the secretion of physiologic CXCL12 that Ray et al. showed to form dimers in physiologic conditions and high concentrations that more efficiently activate pathways leading to pathology and malignancy and inhibit chemotaxis (32, 62, 63). Recombinant CXCL12, specifically purified monomeric CXCL12 (32, 62, 64), has demonstrated its protective effects against myocardial infarction during ischemia/reperfusion injury (27, 31, 32, 65–69). Veldkamp et al. also suggested that low concentrations of monomeric CXCL12 stimulates chemotaxis (63) and is the active form for chemotaxis that confers cardioprotection during I/R myocardial infarction (32). Monomeric CXCL12 is secreted by both mammalian cells and bacteria (62) and commercially available in recombinant, purified form, as used in our treatment injections.

Because of the high risk of coagulopathy and mortality described in the bilateral I/R model (38, 70), we chose to replicate the 60-min unilateral renal I/R model, with a minor increase to 65 min. Since only one kidney was damaged, minimal changes could be detected in renal function clinical pathology endpoints (*p* > 0.05) (Figure 5) due to compensation by the untouched contralateral kidney. More recently, a 90-min unilateral renal I/R model has been described (71) as well as other protocols using various combinations of I/R and delayed contralateral nephrectomy (72) that may better detect significant changes in renal function in blood and urine samples for future pre-clinical models. Interestingly, elevation of the renal biomarker SDMA >14 ug/dL was observed in 11/30 cats at baseline (with 4/30 cats having SDMA ≥18 ug/dL at baseline) and fluctuated regardless of treatment group, and 3/6 cats in the uninjured Control group had SDMA >14 at the end of the study (Pre-clinical Supplementary Table 4). Recently, Brans et al. suggested that SDMA readings using the cut off 18 ug/dL rather than 14 ug/dL may be a more appropriate indicator of early kidney disease that could avoid false positives (50). We also measured changes in body weight (Figure 5G) and USG (Figure 5H) for all groups when compared to Day 70 post-injury (i.e., maximum injury), just prior to Treatment injection, but there was no significant difference among groups.

We focused our evaluation on the quantitative analysis of treatment effects on fibrosis in the injured kidney cortico-medullary junction (CMJ), where lesions should be most severe (38). Our I/R model significantly increased tubulointerstitial collagen content in the injured renal CMJ as quantified on Masson's Trichrome stain and validated with PSR-POL. Both the Mid and High doses of CXCL12 injected 70 days post-injury significantly decreased tubulointerstitial collagen content using kidney tissues stained with both Masson's Trichrome (*p* < 0.05 vs. untreated) and PSR-POL (*p* < 0.01 vs. untreated) 4 months post-injection. Although statistical results of pairwise comparisons were slightly different between Masson's Trichrome stain and PSR-POL, the overall CXCL12 Mid and High dose treatment effects on fibrosis were consistent despite using the two different quantification methods and analyses for collagen.

Normal tissue homeostasis is maintained through a delicate balance of collagen production, deposition, and degradation in the extracellular matrix (ECM) (73). Irreversible fibrosis results when the tissue microenvironment favors ECM production and deposition over degradation and remodeling. Analysis of collagen fiber parameters, such as length, width, angularity, and straightness, in normal and pathologic disease states may help unlock the conditions that favor irreversible fibrosis (40, 43, 44, 46), and this method of evaluating collagen has been validated by Caetano et al. as comparable to the gold standard hydroxyproline assay (45). Increased collagen fiber width in the tumor microenvironment is a powerful negative prognostic indicator associated with reduced 5-year survival that was validated in two separate gastric cancer cohorts of 225 and 151 cancer patients (42). Our I/R model significantly increased collagen fiber width in the injured renal CMJ as analyzed with PSR-POL. Both the Mid and High doses of recombinant CXCL12 injected 70 days post-injury significantly decreased collagen fiber width (*p* < 0.001 vs. untreated) at 4 months post-injection. Incorporating segmentation software-generated analyses of collagen fiber histomorphometry, such as CT-FIRE, and in particular, evaluating the collagen fiber parameter of width, could more accurately depict which microenvironments may favor irreversible fibrosis over collagen degradation and remodeling that may impact prognosis.

A proposed mechanism for the observed attenuation in I/R-induced renal fibrosis and collagen fiber widening may be early, short-term CXCL12 treatment effects on collagenases and collagen cross-linking enzymes as suggested by our small study findings. MMP-1 is a collagenase that degrades fibrillar collagen and disrupts its 3-dimensional triple-helical structure to increase exposure and susceptibility to further degradation by other proteases (74). CXCL12 stimulation has been shown to have early, dose-dependent increases in MMP-1 production (75–77), and CXCL12 signaling and crosstalk with MMP-1 both stimulate stem cell migration (78). Early changes (increases) in MMP-1 concentrations post-CXCL12 injection may play a role in the attenuation of renal fibrosis with CXCL12 treatment, but our small sample size (*n* = 1) precludes us from making this conclusion. LOXL-2 crosslinks collagens and elastin resulting in increased ECM stiffness (79) and has been associated with renal fibrosis and pathology (80, 81). ECM scaffold treatment with anti-LOXL2 antibodies demonstrated a decrease in average collagen fiber width (82). We suspect that early changes (decreases) in LOXL-2 concentrations post-CXCL12 injection may also play a role in re-establishing a more normal ECM environment favoring collagen fibers of normal width that are potentially more susceptible to remodeling. However, the small sample size of our study evaluating early changes in MMP-1 and LOXL-2 concentrations (*n* = 1/early time point) is a major limitation. More studies that include local concentrations measured at baseline (just prior to treatment injection), more time points, and larger sample sizes are needed to investigate these and other possible mechanisms with statistical significance.

In the pilot study, recombinant CXCL12 treatment for cats with early CKD was safe and feasible in a general practice setting, and we measured changes in kidney disease stage progression, USG, serum creatinine, and SDMA in both groups. At the study end, there were no treated cats that advanced to a higher stage of CKD. Most cats who successfully met our inclusion criteria and requirements were placed into the Treatment group early in the study, thus, were not randomized. All cats who were enrolled in the study were treated for any pre-existing conditions. The 2 cats in the Control group with suspected hypertrophic cardiomyopathy (Cats 5 and 6) were considered stable with their instituted therapy. SDMA ELISA kits were used as an alternative to the proprietary IDEXX Laboratory assay due to geographic inaccessibility in Indonesia. Despite the aforementioned limitations, small sample size without randomization, and missing data points, our study supports future studies to evaluate the potential use and efficacy of CXCL12 therapy in a clinical setting.

All cats in both pilot study groups were fed dry Royal Canin Renal Special prescription diet at the beginning and throughout the duration of the study, but similar to what veterinarians experience in other countries, we cannot guarantee that was the only food the cats ate, with the exception of 2 clinic cats in the Treatment Group (Cats 1 and 2). These 2 cats received no other standard of care treatment other than prescription renal food, similar to some cat owners who are unable to medicate their cats. Although the consumption of prescription renal food has been shown to slow the increase in serum creatinine and help maintain lean muscle mass, no effects on urine concentration ability or SDMA have been reported (83).

The combined data from our pre-clinical and pilot studies suggest that intra-renal injection of recombinant CXCL12 may be a novel treatment for chronic renal fibrosis in cats with the potential for future use in other veterinary species and translational applications in humans (Figure 1). However, more controlled studies with larger sample sizes and randomization will be needed to evaluate treatment efficacy with statistical significance in a clinical setting. The ideal candidates for CXCL12 therapy may be in the early stages of kidney disease, which can have subtle or no clinical signs, therefore, elevating the value of routine wellness screening and early disease detection. Further studies are needed to identify CXCL12 formulations that may increase the efficacy of this therapy to treat more advanced kidney disease and to fully elucidate the mechanism, tissue and systemic dispersion, and duration of effect.

## Data Availability Statement

The original contributions generated in the study are included in the article/Supplementary Material, further inquiries can be directed to the corresponding author.

## Ethics Statement

The animal study was reviewed and approved by Institutional Animal Care and Use Committee (IACUC) at the Wake Forest University School of Medicine. Written informed consent was obtained from the owners for the participation of their animals in this study.

## Author Contributions

JB, SL, RM, DS, CK, IS, WK, MP, SM, GB, and JW contributed to the conception and design of the study. JB, SL, RM, DS, JF, CK, IS, WK, MP, SM, and JW contributed to the data acquisition, analysis, and interpretation of the work. JB and JF performed the statistical analyses. JB and JW wrote the first draft of the manuscript, and all authors contributed to revisions, read and approved the submitted version, and agreed to be accountable for the work. All authors contributed to the article and approved the submitted version.

## Conflict of Interest

JW is an inventor on patent rights related to this work owned by Wake Forest University Health Sciences. The patents, whose value may be affected by publication, have the potential to generate royalty income in which the inventors would share. The remaining authors declare that the research was conducted in the absence of any commercial or financial relationships that could be construed as a potential conflict of interest.
