# Supplementary material for: Chemokine Therapy in Cats With Experimental Renal Fibrosis and in a Kidney Disease Pilot Study
Source: Front Vet Sci. 2021 Mar 4;8:646087. doi: 10.3389/fvets.2021.646087 (PMC7969654; doi:10.3389/fvets.2021.646087)
Supplement: Supplementary file 1 [file Data_Sheet_1.PDF]

Preclinical Supplementary Material Table of Contents:

Supplemental Figure 1. Changes Renal Concentrations of CXCL12, MMP1, and LOXL2 – page 2-3

Supplemental Table 1. Urine Specific Gravity (Preclinical Study) – page 4

Supplemental Table 2. Body Weight (kg) (Preclinical Study) – page 5

Supplemental Table 3. Serum Creatinine (mg/dL) (Preclinical Study) – page 6

Supplemental Table 4. SDMA (ug/dL) (Preclinical Study) – page 7

Supplemental Table 5. Serum BUN (mg/dL) (Preclinical Study) – page 8

Supplemental Table 6. Serum Phosphorus (mg/dL) (Preclinical Study) – page 9

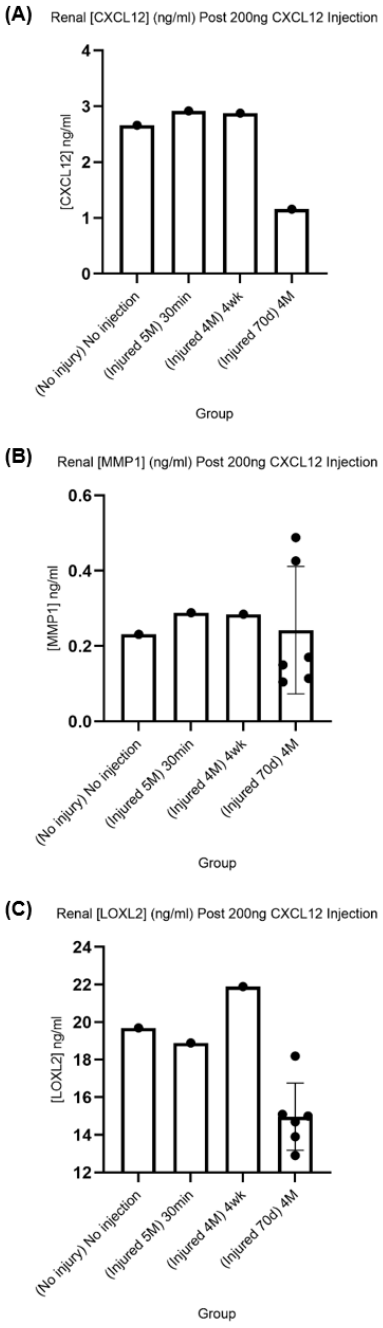

Supplemental Figure 1. Changes in Renal Concentrations of CXCL12, MMP1, and LOXL2. Renal concentrations at 30 minutes, 4 weeks, and 4 months post 200ng CXCL12 injection. (A) Renal concentration of CXCL12 at 30 minutes post-injection (after 5 months I/R injury), 4 weeks post-injection (after 4 months I/R injury), and 4 months post-injection (after 70 days I/R injury) showed no significant difference. n=1/group. (B) Renal concentration of MMP1 at 30 minutes (n=1), 4 weeks (n=1), and 4

months post-injection (n=6) showed no significant difference. (C) Renal concentration of LOXL2 at 30 minutes (n=1), 4 weeks (n=1), and 4 months post-injection (n=6) showed no significant difference. Values are dot plot and mean +/- SD.

Supplemental Table 1. Urine Specific Gravity (Preclinical Study)

Group	Base	D70	M1	M2	M3	M4
Carrier 1	1.032	1.053	1.026	1.026	1.047	1.050
Carrier 2	1.042	1.052	1.030	1.056	1.051	1.051
Carrier 3	1.041	1.031	1.018	1.042	1.052	1.037
Carrier 4	1.045	1.049	1.046	1.050	1.052	1.048
Carrier 5	1.045	1.051	1.030	1.040	1.040	1.035
Carrier 6	1.035	1.053	1.045	1.045	1.050	1.044
Low 1	1.060	1.050	1.047	1.048		1.051
Low 2	1.054	1.044	1.050	1.045	1.049	1.054
Low 3	1.032	1.010	1.011	1.010	1.020	1.024
Low 4	1.029	1.049	1.046	1.040	1.030	1.039
Low 5	1.047	1.032	1.049	1.038	1.046	1.047
Low 6	1.054	1.053	1.052	1.046	1.055	1.057
Mid 1	1.044	1.050	1.049	1.037	1.031	1.051
Mid 2	1.021	1.025	1.041	1.014	1.040	1.053
Mid 3	1.036	1.020	1.045	1.043	1.035	1.041
Mid 4	1.038	1.017	1.025	1.032	1.031	1.045
Mid 5	1.046	1.045	1.039	1.042	1.043	1.059
Mid 6	1.040	1.051	1.046	1.045	1.047	1.025
High 1	1.042	1.050	1.007	1.041	1.030	1.036
High 2	1.028	1.040	1.042	1.032	1.051	1.045
High 3	1.043	1.046	1.030	1.010	1.041	1.048
High 4	1.056	1.042	1.049	1.046	1.047	1.043
High 5	1.044	1.056	1.042	1.050	1.053	1.045
High 6	1.050	1.056	1.054	1.049	1.054	1.031
Control 1	1.041	1.050	1.045	1.037	1.049	1.059
Control 2	1.045	1.055	1.028	1.041	1.042	1.050
Control 3	1.034	1.032	1.036	1.042	1.040	1.046
Control 4	1.044	1.041	1.035	1.034	1.046	1.038
Control 5	1.040	1.046	1.044	1.042	1.048	1.056
Control 6	1.045	1.051	1.045	1.041	1.036	1.045

Supplemental Table 2. Body Weight (kg) (Preclinical Study)

Group	Base	D70	M1	M2	M3	M4
Carrier 1	2.3	2.3	2.4	2.2	2.3	2.3
Carrier 2	3.5	3.3	3.2	3.1	3	2.9
Carrier 3	3.3	3.3	3.7	3.3	3.6	3.7
Carrier 4	3.7	3.9	3.9	3.9	3.8	3.8
Carrier 5	3.1	3.3	3.3	3.3	3.4	3.9
Carrier 6	2.5	3.1	3.4	3.5	3.5	3.7
Low 1	2.7	3	3.3	3.3	3.3	3.4
Low 2	3.7	3.4	3.2	3.4	3.3	3.3
Low 3	2.8	2.9	3.1	2.9	3.1	3.1
Low 4	2.8	3.1	3.2	3.2	3.3	3.4
Low 5	2.5	2.8	2.6	2.8	2.8	2.7
Low 6	3.1	3.1	2.8	2.8	2.7	2.6
Mid 1	2.7	3	3.2	3.3	3.2	3.4
Mid 2	2.7	2.7	3.1	3.5	3.8	3.8
Mid 3	2.8	3	2.8	2.7	2.8	3
Mid 4	2.8	2.9	2.9	2.9	2.8	
Mid 5	3.1	3.1	3.2	3.1	3.4	3.2
Mid 6	3.1	3.4	3.4	3.2	3.2	3.1
High 1	3	3.5	3.2	3.4	3.2	3
High 2	3	3.8	3	3.1	2.9	3.1
High 3	3.1	3.6	3.4	3.3	2.9	3
High 4	2.9	3.2	3	3.2	3.1	3
High 5	2.9	3.2	3.1	3.2	2.9	3.1
High 6	3	3.4	3.1	3	2.8	2.8
Control 1	3.2	3.3	3.1	3.3	3.1	3.1
Control 2	2.8	2.8	3	2.8	2.8	2.7
Control 3	2.9	2.5	2.4	2.4	2.4	2.3
Control 4	3.2	3.4	3.1	3.1	2.9	3
Control 5	2.8	2.8	3	3	2.9	2.9
Control 6	3.3	3.5	3.2	3.2	3.1	2.9

Supplemental Table 3. Serum Creatinine (mg/dL) (Preclinical Study)

Group	Base	D70	M1	M2	M3	M4
Carrier 1	0.8	0.7	0.7	0.9	0.9	0.9
Carrier 2	1	1	1.1	1.3	1.2	1.4
Carrier 3	1.0	0.9	1.1	1.3	1.4	1.3
Carrier 4	1	0.9	1.4	1.1	1	1.2
Carrier 5	1.2	1	1.5	1.5	1.4	1.2
Carrier 6	0.9	0.8	1	1.1	1.2	1.2
Low 1	0.8	1	1	1	0.9	1.2
Low 2	1.7	1.5	1.4	1.4	1.5	1.6
Low 3	1.0	1.3	1.5	1.4	1.6	1.5
Low 4	0.9	1	0.9	1.1	1	1.3
Low 5	0.8	1.2	1.2	1	1	1.2
Low 6	0.8	0.9	0.9	0.7	0.8	1
Mid 1	1	1.1	1.2	1.2	1.2	1.2
Mid 2	1.1	0.9	0.9	1	1.1	1.9
Mid 3	0.7	0.8	1	0.8	1.2	1.2
Mid 4	0.9	1.1	0.9	0.9	1.3	1.5
Mid 5	0.7	1	0.9	1.2	0.9	0.9
Mid 6	1.0	1.1	1	1.4	1.3	1.2
High 1	1	1	0.9	1.2	1.2	1.1
High 2	1.0	1	1.5	1.3	1.5	1.2
High 3	0.9	0.9	1.3	1	1	1.2
High 4	0.9	1.3	0.7	0.8	0.9	0.9
High 5	0.9	0.8	0.9	1.1	1.1	1.3
High 6	0.9	1	0.9	1.1	1.1	1
Control 1	1.0	1.1	1.1	1.1	1.1	1.6
Control 2	1.0	1	0.8	0.9	0.9	0.9
Control 3	1.0	1.1	1	1.3	1.1	1.2
Control 4	1.1	1.3	1.2	1.3	1.4	1.3
Control 5	1.0	1.1	1.2	1.3	1.1	1.7
Control 6	1.2	1.2	1	0.9	0.8	1.2

Supplemental Table 4. SDMA (ug/dL) (Preclinical Study)

Group	Base	D70	M1	M2	M3	M4
Carrier 1	9	13	12	10	12	12
Carrier 2	9	10	7	8	5	11
Carrier 3	11	15	15	13	17	12
Carrier 4	14	15	13	11	13	15
Carrier 5	13	15	13	13	16	14
Carrier 6	12	10	13	9	13	13
Low 1	13	12	16	14	14	16
Low 2	17	15	15	15	16	15
Low 3	11	14	17	16	17	13
Low 4	13	15	18	17	15	16
Low 5	11	12	14	13	13	15
Low 6	13	15	15	16	15	14
Mid 1	18	12	17	18	17	19
Mid 2	19	18	11	15	15	18
Mid 3	10	13	10	9	11	12
Mid 4	14	13	13	12	14	14
Mid 5	21	16	11	14	16	15
Mid 6	17	14	14	12	14	13
High 1	13	10	7	11	11	12
High 2	10	11	9	12	13	11
High 3	15	8	10	13	10	14
High 4	13	15	11	10	11	13
High 5	11	11	11	15	9	12
High 6	16	16	14	17	14	14
Control 1	17	12	16	9	11	15
Control 2	18	11	11	15	15	17
Control 3	15	10	12	13	14	16
Control 4	11	10	12	10	12	10
Control 5	12	10	15	10	10	14
Control 6	16	13	12	12	12	13

Supplemental Table 5. Serum BUN (mg/dL) (Preclinical Study)

Group	Base	D70	M1	M2	M3	M4
Carrier 1	21	22	24	23	22	20
Carrier 2	22	22	20	23	21	23
Carrier 3	26	28	28	20	22	21
Carrier 4	26	26	30	24	21	21
Carrier 5	29	26	26	27	26	19
Carrier 6	23	25	27	28	25	25
Low 1	27	27	29	25	24	24
Low 2	27	28	34	22	24	22
Low 3	25	28	34	23	27	23
Low 4	29	27	25	27	25	26
Low 5	26	27	25	23	23	22
Low 6	35	25	26	26	26	28
Mid 1	23	28	29	27	29	27
Mid 2	24	26	29	25	31	29
Mid 3	25	21	18	26	18	18
Mid 4	25	24	19	23	18	22
Mid 5	30	31	22	30	27	34
Mid 6	22	30	31	30	27	17
High 1	21	28	21	25	24	24
High 2	21	29	24	25	29	26
High 3	22	26	22	22	22	28
High 4	25	45	25	23	24	28
High 5	25	31	22	27	25	27
High 6	26	30	20	23	22	22
Control 1	25	29	24	26	26	28
Control 2	29	28	20	24	27	29
Control 3	28	30	26	24	28	28
Control 4	34	32	26	25	27	28
Control 5	22	28	25	20	18	19
Control 6	26	27	27	25	25	23

Supplemental Table 6. Serum Phosphorus (mg/dL) (Preclinical Study)

Group	Base	D70	M1	M2	M3	M4
Carrier 1	4.3	3.8	4.0	4.5	3.8	3.8
Carrier 2	6.2	4.5	4.2	4.6	4.9	4.1
Carrier 3	6.3	5.5	5.7	5.4	6.5	5.5
Carrier 4	6.4	5.2	7.2	5.5	5.4	4.7
Carrier 5	6.6	5.4	7.3	5.1	8.5	5.8
Carrier 6	5.8	5.8	6.1	5.9	5.6	5.4
Low 1	6.2	6.7	5.6	5.7	5.4	5.9
Low 2	7.0	5.8	6.0	5.9	5.7	6.0
Low 3	5.6	6.0	5.1	5.3	5.5	5.4
Low 4	6.5	5.5	5.9	5.8	5.3	5.7
Low 5	7.3	5.8	6.0	5.2	4.6	5.2
Low 6	7.2	5.8	4.7	4.7	4.5	4.8
Mid 1	7.2	7.1	7.2	6.5	6.3	5.5
Mid 2	6.5	6.8	7.8	6.6	6.8	8.5
Mid 3	8.3	6.2	6.3	6.7	6.7	4.7
Mid 4	7.4	5.2	5.4	5.2	5.7	6.1
Mid 5	7.0	5.5	5.5	6.9	6.5	5.4
Mid 6	7.8	7.5	6.2	6.0	6.9	5.2
High 1	8.0	8.0	7.6	5.4	5.7	5.4
High 2	7.8	7.6	6.7	6.2	5.5	6.0
High 3	7.0	6.1	7.7	6.7	5.0	5.4
High 4	7.4	6.0	5.9	4.9	4.5	4.4
High 5	7.7	7.8	6.8	6.1	5.7	5.2
High 6	7.4	7.3	6.3	6.0	5.7	5.4
Control 1	6.8	6.6	5.9	5.8	5.5	6.6
Control 2	7.3	6.2	6.5	5.5	5.6	5.1
Control 3	6.8	6.3	5.1	4.7	4.4	4.3
Control 4	7.7	6.9	5.8	6.0	5.0	7.0
Control 5	6.2	6.6	6.0	4.6	5.5	3.6
Control 6	7.0	5.5	6.1	5.5	5.8	5.3
