# Supplementary material for: Chemokine Therapy in Cats With Experimental Renal Fibrosis and in a Kidney Disease Pilot Study
Source: Front Vet Sci. 2021 Mar 4;8:646087. doi: 10.3389/fvets.2021.646087 (PMC7969654; doi:10.3389/fvets.2021.646087)
Supplement: Supplementary file 2 [file Data_Sheet_2.PDF]

Pilot Supplementary Material Table of Contents:

Supplemental Table 1. Descriptive Statistics for Renal Function Clinical Pathology Endpoints Presented as Median (Range) – page 2

Supplemental Table 2. CKD Stage (Pilot Study) – page 3

Supplemental Table 3. Urine Specific Gravity highlighting 1.035 or higher (Pilot Study) – page 3

Supplemental Table 4. Serum Creatinine (mg/dL) highlighting 1.4 or less (Pilot Study) – page 4

Supplemental Table 5. SDMA (ug/dL) highlighting 14 or less (Pilot Study) – page 4

Supplemental Figure 1. Pilot Study Summary Statistics for CKD stage progression, USG, and SDMA – page 5

Supplemental Table 1. Pilot study descriptive statistics for renal function clinical pathology endpoints presented as median (range)

	Baseline	Month 1	Month 2	Month 3	Month 4	Month 5	Month 6	Month 7	Month 8	Month 9	Month 10	Month 11	Month 12
**CKD stage**													
**Control**	2 (1-2)	2 (1-2)	2 (1-2)	2 (0-2)	2 (1-3)	2 (1-3)	1 (0-2)	2 (1-2)	2 (1-3)	2 (1-3)	2 (0-2)	2 (1-2)	3 (2-3)
**n**	7	7	7	7	7	7	7	6	6	6	5	4	3
**Treatment**	2 (1-2)	1 (0-2)	1 (1-2)	2 (1-2)	1 (0-2)	1 (0-2)	2 (1-2)	1 (0-2)	1 (1-2)	2 (1-2)	1 (0-2)	2 (1-2)	1 (0-2)
**n**	7	7	7	7	7	7	7	7	7	7	6	5	5
**USG (>1.035)**													
**Control**	1.015 (1.010-1.020)	1.020 (1.010-1.030)	1.010 (1.010-1.030)	1.020 (1.010-1.050)	1.025 (1.010-1.050)	1.020 (1.010-1.033)	1.018 (1.010-1.040)	1.025 (1.010-1.037)	1.015 (1.015-1.040)	1.017 (1.010-1.030)	1.020 (1.010-1.035)	1.018 (1.010-1.020	1.015 (1.010-1.020)
**n**	7	6	5	7	6	7	6	6	5	4	5	4	3
**Treatment**	1.020 (1.010-1.034)	1.030 (1.020-1.040)	1.015 (1.010-1.040)	1.025 (1.020-1.030)	1.025 (1.010-1.050)	1.025 (1.012-1.050)	1.020 (1.020-1.030)	1.030 (1.010-1.047)	1.030 (1.010-1.040)	1.020 (1.020-1.031)	1.033 (1.020-1.045)	1.030 (1.030-1.045)	1.049 (1.040-1.058)
**n**	7	5	7	5	5	6	4	7	5	3	4	4	2
**Serum creatinine (<1.6 mg/dL)**													
**Control**	1.8 (1.3-2.4)	1.7 (1.2-2.6)	1.8 (1.4-2.4)	1.8 (1.4-2.5)	2.2 (1.6-4.3)	1.7 (1.4-3.9)	1.8 (1.3-2.3)	1.9 (1.2-2.1)	2 (1.4-2.9)	2.1 (1.3-3.2)	1.6 (1.1-2.6)	1.8 (1.2-2.4)	2.9 (1.8-3.4)
**n**	7	7	7	7	7	7	7	6	6	6	5	4	3
**Treatment**	1.7 (1.4-2.1)	1.5 (1.3-2.6)	1.3 (.9-2.4)	1.7 (1-2.2)	1.8 (1.3-2.8)	1.3 (1-2.3)	1.6 (1.2-2)	1.7 (1.1-2.1)	1.7 (1.2-2.4)	1.8 (.9-2.6)	1.7 (.6-2.8)	1.8 (1.3-2.1)	1.5 (1.3-2.1)
**n**	7	7	7	7	7	7	7	7	7	7	6	5	5
**Body weight (kg)**													
**Control**	3 (2.5-6.3)	3.2 (2.6-6.1)	3.3 (2.7-6.2)	3.3 (2.8-6.4)	3.1 (2.8-6.4)	3.2 (2.8-6.4)	3 (2.8-6.2)	3.9 (2.6-6.5)	3.9 (2.6-6.5)	3.9 (2.6-6.4)	5 (2.6-6.4)	5.4 (2.6-6.4)	4.8 (2.6-5.9)
**n**	7	7	7	7	7	7	7	6	6	6	5	4	3
**Treatment**	4.7 (3.4-5.9)	4.8 (3.1-5.9)	5 (2.9-5.8)	4.7 (2.8-5.6)	4.4 (2.8-5.6)	4.8 (2.8-5.7)	4.9 (2.8-5.7)	4.5 (2.6-5.7)	4.7 (2.7-5.8)	4.7 (2.4-6)	4.7 (2.4-5.5)	4.7 (4.4-5.6)	4.8 (4.6-5.6)
**n**	7	7	7	7	7	7	7	7	7	7	6	5	5
**SDMA (14-18 ug/dL or less)**													
**Control**	14 (13-27)	16 (2-20)	14 (6-67)	13 (7-53)	16 (8-34)	17 (12-43)	15 (6-32)	16 (14-20)	14 (11-18)	14 (12-18)	21 (13-22)	22 (16-32)	13 (5-20)
**n**	7	7	7	7	7	7	6	5	5	5	3	3	2
**Treatment**	20 (6-24)	11 (6-44)	10 (5-31)	6 (2-15)	13 (6-16)	15 (11-24)	17 (10-54)	12 (4-17)	15 (11-22)	14 (3-21)	13 (5-15)	13 (13-17)	14 (11-24)
**n**	7	7	7	7	7	7	7	7	7	6	5	3	3
**Serum BUN (20-30 mg/dL)**													
**Control**	70.3 (43-90)	61.3 (47-150.1)	58.1 (36-108)	66.9 (41.1-147.8)	71.7 (45.2-120)	61.9 (40-104)	56.8 (41-96)	70.2 (37.4-94.7)	63.2 (54.5-151)	64.4 (41-163.9)	45 (28.7-130.5)	77.4 (36.6-135.7)	91.5 (55.6-91.5)
**n**	7	7	7	7	7	7	7	6	6	6	5	4	3
**Treatment**	51 (39.6-87)	52 (35-70.2)	53.1 (27-80.4)	53 (39.1-90)	55.1 (36-74)	65 (40-79)	58 (47-87.8)	51.5 (45.4-87.8)	61 (49.8-72.3)	55 (39.7-123)	44 (39-83.8)	58.7 (45.3-59.4)	51.4 (44.5-60)
**n**	7	7	7	7	7	7	7	7	7	7	6	5	5
**Serum phosphorus (2.7-7.6 mg/dL)**													
**Control**	4.3 (2.9-5.3)	4.8 (2.6-6)	4.8 (3.2-5.8)	5.2 (3.2-7)	4.4 (2.8-7.4)	4.7 (2.9-6.8)	4.1 (3-5.9)	5.1 (3.3-6.5)	4.4 (3-5.9)	5.2 (3.6-7.6)	5.2 (4-6.5)	5.1 (3.6-6.7)	5.5 (3.5-6)
**n**	7	7	7	7	7	7	7	6	6	6	5	4	3
**Treatment**	4.5 (3-4.8)	5.5 (4.5-6.1)	6 (4.5-6.5)	4.1 (3.3-6.2)	5 (3.1-6)	6.2 (4-7.5)	5 (3.8-6)	5.4 (4.4-6.7)	4.3 (3.5-6)	4.9 (4.2-7.5)	5 (3.2-6.4)	4.3 (3.7-5.3)	4.7 (3.2-6.2)
**n**	7	7	7	7	7	7	7	7	7	7	6	5	5

Supplemental Table 2. CKD Stage (Pilot Study)

Control	Base	1m	2m	3m	4m	5m	6m	7m	8m	9m	10m	11m	12m
Cat 1	1	2	2	1	2	1	1	1	1	2	2	1	2
Cat 2	2	2	2	2	3	3	2	2	3	2	2	2	3
Cat 3	2	2	2	2	1	2	1	2	1	2	2	2	3
Cat 4	2	1	1	1	1	2	0	1	1	1	0		
Cat 5	2	2	2	2	2	2	2	2	2	2	2	1	
Cat 6	2	2	2	2	2	2	2	2	2	3			
Cat 7	2	2	2	0	2	2	1						
**Treated**	**Base**	**1m**	**2m**	**3m**	**4m**	**5m**	**6m**	**7m**	**8m**	**9m**	**10m**	**11m**	**12m**
Cat 1	2	0	1	1	1	0	1	1	1	1	2	1	1
Cat 2	2	2	1	1	1	2	2	1	1	2	2	2	2
Cat 3	1	1	1	1	2	1	2	1	1	2	0	1	1
Cat 4	2	2	1	2	2	2	2	1	2	2	1	2	0
Cat 5	1	1	1	2	0	0	1	0	1	2	1		
Cat 6	2	1	2	2	1	1	2	2	1	2	1	2	1
Cat 7	2	2	1	2	2	2	2	2	2	1			

Supplemental Table 3. Urine Specific Gravity highlighting 1.035 or higher (Pilot Study)

Control	Base	1m	2m	3m	4m	5m	6m	7m	8m	9m	10m	11m	12m
Cat 1	1.010	1.015	1.010	1.010	1.010	1.015	1.010	1.010	1.015	1.010	1.010	1.015	1.010
Cat 2	1.015	1.020	1.015	1.020	1.030	1.020	1.015	1.020	1.015	1.020	1.020	1.020	1.020
Cat 3	1.010	1.010	1.030	1.030	1.050	1.020	1.035	1.030	1.040	1.030	1.030	1.010	1.015
Cat 4	1.015	1.020	1.010	1.020	1.043	1.033	1.040	1.037			1.035		
Cat 5	1.010	1.020		1.020	1.020	1.020	1.010	1.030	1.024		1.020	1.020	
Cat 6	1.020	1.030	1.010	1.020	1.020	1.020	1.020	1.020	1.015	1.013			
Cat 7	1.015			1.050		1.010							
**Treated**	**Base**	**1m**	**2m**	**3m**	**4m**	**5m**	**6m**	**7m**	**8m**	**9m**	**10m**	**11m**	**12m**
Cat 1	1.010	1.040	1.020	1.025	1.025	1.040		1.020					
Cat 2	1.020	1.020	1.020	1.030	1.020	1.030		1.030				1.030	
Cat 3	1.034		1.010				1.020	1.045	1.040		1.045	1.045	
Cat 4	1.020	1.030	1.015	1.020		1.015		1.040	1.010	1.020	1.045	1.030	1.040
Cat 5	1.017	1.030	1.015		1.050	1.050	1.030	1.047	1.030	1.020	1.020		
Cat 6	1.020		1.010	1.020	1.045	1.020	1.020	1.010	1.040		1.020	1.030	1.058
Cat 7	1.015	1.020	1.040	1.030	1.010	1.012	1.020	1.015	1.010	1.031			

Supplemental Table 4. Serum Creatinine (mg/dL) highlighting <1.6 (Pilot Study)

Control	Base	1m	2m	3m	4m	5m	6m	7m	8m	9m	10m	11m	12m
Cat 1	1.3	1.6	1.7	1.4	2.3	1.4	1.3	1.2	1.5	1.6	1.6	1.2	1.8
Cat 2	2.2	2.6	2.4	2.5	4.3	3.9	2.3	2.1	2.9	2.5	1.8	2.4	3.4
Cat 3	2.0	1.8	2.2	2.2	2.2	1.6	1.9	1.9	2.1	2.1	2.6	2.3	2.9
Cat 4	1.8	1.2	1.4	1.5	1.6	1.7	1.4	1.7	1.4	1.3	1.1		
Cat 5	1.7	1.6	1.8	2.1	2.1	1.7	1.8	1.8	2.6	2.0	1.6	1.3	
Cat 6	2.4	2.6	2.2	1.8	2.8	2.0	2.1	1.9	1.9	3.2			
Cat 7	1.8	1.7	1.8	1.5	1.8	2.5	1.5						
**Treated**	**Base**	**1m**	**2m**	**3m**	**4m**	**5m**	**6m**	**7m**	**8m**	**9m**	**10m**	**11m**	**12m**
Cat 1	1.6	1.3	0.9	1	1.4	1.2	1.2	1.1	1.2	1.5	1.8	1.3	1.5
Cat 2	2	1.6	1.1	1.4	1.3	1.6	1.6	1.3	1.5	2.1	2.8	2	2.1
Cat 3	1.4	1.5	1.3	1.2	1.8	1.0	1.7	1.7	2.3	1.8	1.5	2.1	1.3
Cat 4	1.7	1.6	1.0	2.2	2.1	2.3	1.6	1.7	2.2	2.0	1.9	1.8	1.5
Cat 5	1.5	1.4	1.5	1.7	1.3	1.3	1.3	1.4	1.4	2.6	0.6		
Cat 6	1.7	1.5	2.4	2.2	2.5	1.3	1.6	2.0	2.4	1.6	0.8	1.7	1.8
Cat 7	2.1	2.6	2.0	2.1	2.8	2.1	2.0	2.1	1.7	0.9			

Supplemental Table 5. SDMA (ug/dL) highlighting 14 or less (Pilot Study)

Control	Base	1m	2m	3m	4m	5m	6m	7m	8m	9m	10m	11m	12m
Cat 1	14	16	16	12	12	12	6	14	14	14	13	32	20
Cat 2	14	17	14	11	16	17	8	18	18	18	21	16	5
Cat 3	14	20	67	7	8	12	32	20	14	17	22	22	
Cat 4	18	5	6	13	11	14	16	16	11	12			
Cat 5	18	5	6	34	34	31	15	15	14	13			
Cat 6	13	20	16	53	32	24							
Cat 7	27	2	6	19	18	43	14						
**Treated**	**Base**	**1m**	**2m**	**3m**	**4m**	**5m**	**6m**	**7m**	**8m**	**9m**	**10m**	**11m**	**12m**
Cat 1	14	12	10	6	12	11	15	14	22	3	5	17	11
Cat 2	15	11	9	5	16	13	11	11	21	7	10	13	24
Cat 3	22	11	28	15	13	24	54	4	11	21	13	13	14
Cat 4	24	14	18	6	6	17	30	14	16	17	15		
Cat 5	20	6	5	2	15	16	10	12	15	13			
Cat 6	22	44	31	14	11	11	21	11	12	15	13		
Cat 7	6	6	9	11	14	15	17	17	15				

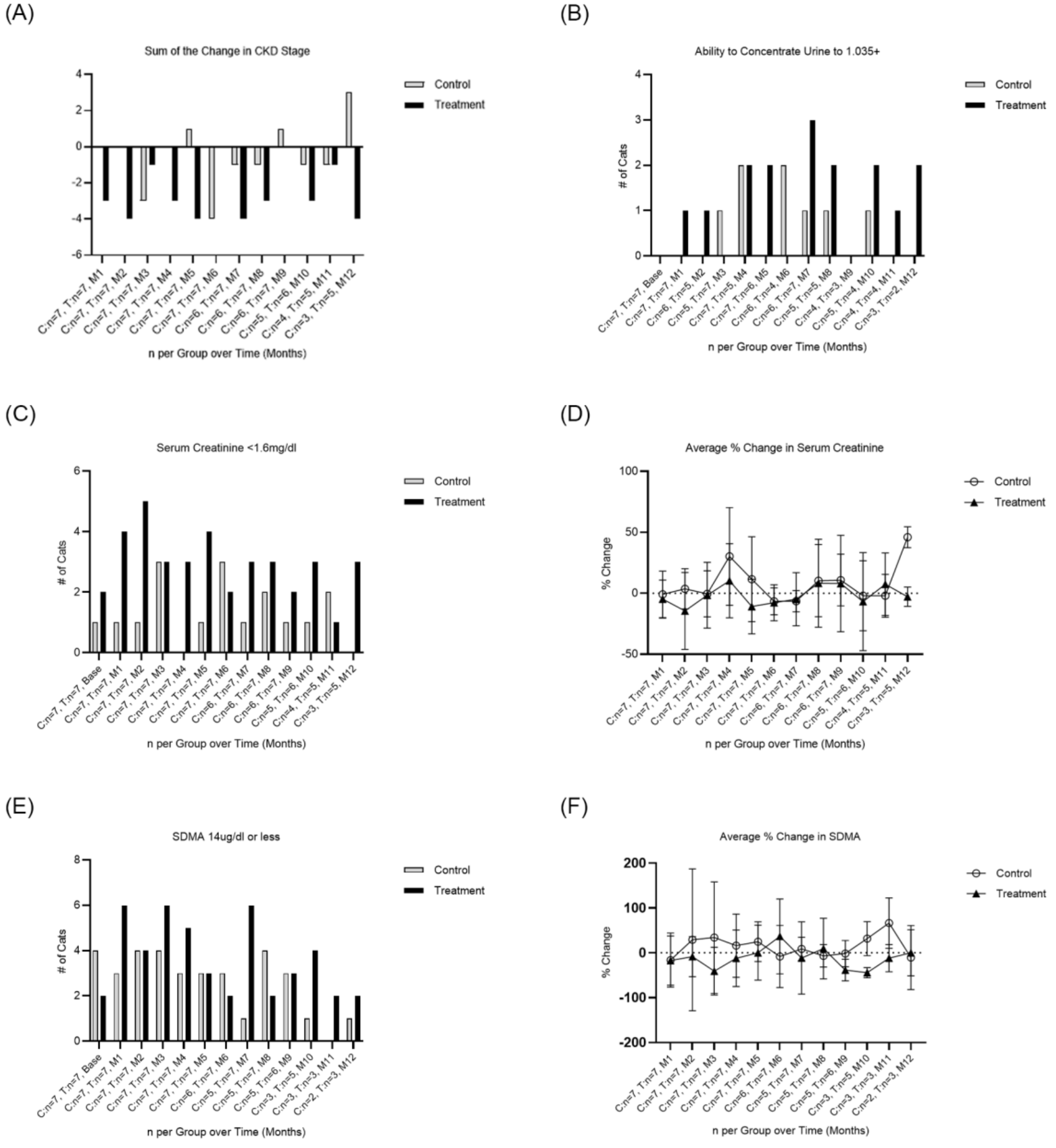

Supplemental Figure 1. Pilot study summary statistics for CKD stage progression, USG, and SDMA with CXCL12 treatment. (A) Sum of the change in CKD stage for each group over time with n specified for Control (C) and Treatment (T) groups at each time point. (B) # of cats able to concentrate urine to USG 1.035 or higher. (C-D) # of cats with serum creatinine <1.6mg/dL and Average % change in creatinine over time compared to baseline. (E-F) # of cats with serum SDMA 14ug/dL or less and Average % change in SDMA.
